# Phytochemicals and REDOX Modulation: Molecular Mechanisms, Clinical Relevance, and Therapeutic Perspectives

**DOI:** 10.3390/antiox15020272

**Published:** 2026-02-22

**Authors:** Desh Deepak Singh, Dharmendra Kumar Yadav, Dongyun Shin

**Affiliations:** 1 Amity Institute of Biotechnology, Amity University Rajasthan, Jaipur 303002, India; ddsbms@gmail.com; 2 College of Pharmacy, Gachon University, Hambakmoeiro 191, Yeonsu-gu, Incheon 21924, Republic of Korea

**Keywords:** oxidative stress, antioxidant phytochemicals, polyphenols, flavonoids, carotenoids

## Abstract

Oxidative stress and redox (REDOX) imbalance play a key role in the development of many chronic and degenerative disorders, including cardiovascular diseases, neurodegenerative conditions, cancer, and age-related illnesses. Beyond causing direct damage to macromolecules, disrupted REDOX signaling affects cellular homeostasis, alters inflammatory responses, and modifies metabolic control, leading to disease onset and progression. Therefore, targeting oxidative pathways offers a promising therapeutic approach for managing chronic diseases. Naturally derived antioxidants, especially phytochemicals such as polyphenols, flavonoids, and carotenoids, have been identified as novel REDOX modulators with diverse biological effects that extend beyond simple free-radical scavenging. This review provides a detailed overview of the molecular mechanisms through which these phytochemicals influence oxidative pathways and exert protective effects on cells. We discuss their relevance in oxidative stress–related diseases, evaluate current clinical evidence regarding their efficacy, and highlight key challenges that limit their clinical application. Special attention is given to the roles of bioavailability, metabolism, and gut microbiota in shaping health outcomes associated with phytochemical consumption. Additionally, we outline emerging strategies to enhance phytochemical efficacy, including synergistic combinations and advanced delivery systems. Overall, this article underscores the potential of phytochemicals as active modulators of REDOX biology, supporting their role in precision nutrition and modern therapeutic approaches.

## 1. Introduction

The primary cause of oxidative stress occurs when a cell produces excessive reactive oxygen and/or nitrogen species (ROS/RNS) through mitochondrial processes relative to its capacity to neutralize these substances using its intrinsic antioxidant systems [1]. Under normal physiological conditions, cells maintain a well-regulated mechanism to control redox states and preserve homeostasis. In addition, redox signaling is involved in numerous processes, including regulation of gene expression and signal transduction, as well as control of immune system function and mitochondrial activity [2]. When excessive free radicals are generated, resulting in oxidative stress, they overwhelm cellular antioxidant defense systems, leading to oxidative damage to lipids, proteins, and nucleic acids. Consequently, both the structural integrity and biological function of the cell are compromised [3].

Numerous studies now demonstrate that oxidative stress resulting from redox dysregulation is a major contributor to the underlying pathology of a wide range of chronic and degenerative diseases [4]. In the case of neurodegenerative disorders such as Alzheimer’s and Parkinson’s diseases, oxidative stress is known to be a contributing factor in disease development. These effects are thought to be mediated through mitochondrial dysfunction, protein misfolding, and neuroinflammation [5]. In addition, oxidative stress plays a central role in the pathophysiology of cardiovascular disease, diabetes, cancer, metabolic syndrome, and inflammatory disorders. These conditions are influenced by altered redox signaling, endothelial dysfunction, genomic instability, hyperproliferative activity, and compromised immune function [6].

Endogenous antioxidant defense systems, such as superoxide dismutases (SODs), catalase, glutathione peroxidases (GPx), and thioredoxin networks, respond to oxidative stress through their antioxidant activities [7]. However, the effectiveness of these defense systems can diminish over time due to factors such as aging, exposure to environmental toxins, and poor nutrition. For this reason, restoring redox balance has become a major focus of interest among healthcare practitioners because of its potential to reduce oxidative damage and modulate signaling pathways involved in disease progression [8].

**Figure 1:**
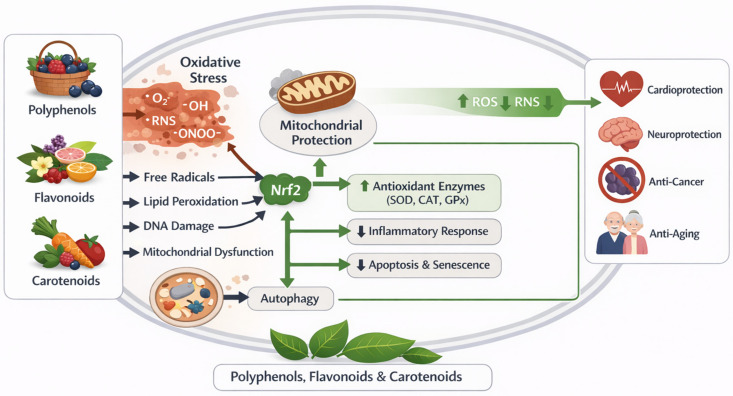
Antioxidant phytochemicals and redox signaling pathway. Created in BioRender. Singh, D. (2026) https://BioRender.com/kb3rzps (accessed on 11 February 2026).

Historically, natural products have provided an abundant source of bioactive compounds. Recently, there has been increasing interest in using plant-based phytochemicals to modulate oxidative stress response pathways [9]. Examples of these compounds include polyphenols, flavonoids, and carotenoids, which belong to structurally diverse classes of phytochemicals found in fruits, vegetables, herbs, and medicinal plants [9]. Unlike synthetic antioxidants that primarily act through direct radical scavenging, phytochemicals exert biological effects via multiple mechanisms beyond direct redox reactions [10]. Recent studies exploring their mechanisms of action indicate that polyphenols and flavonoids are redox-active signaling modulators that influence multiple molecular pathways related to oxidative stress responses [11]. Specifically, polyphenols and flavonoids regulate the activity of transcription factors, including Nrf2, NF-κB, and AP-1, which stimulate the expression of endogenous antioxidant enzymes and inhibit signals produced by pro-inflammatory and pro-oxidant molecules [12]. Additionally, carotenoids not only quench singlet oxygen and lipid peroxyl radicals but also affect membrane integrity, promote mitochondrial function, and facilitate cellular communication during oxidative stress (Figure 1) [13].

Collectively, phytochemicals, including flavonoids, carotenoids, and polyphenols, can contribute to restoring redox homeostasis through coordinated regulatory actions on antioxidant defense systems, inflammatory mediators, and metabolic pathways [14]. The potential therapeutic use of plant-derived antioxidants in chronic disease management is further supported by their relative safety, widespread availability in common foods, and suitability for long-term use [14]. However, significant challenges remain in translating plant-derived antioxidants into clinical practice, including variability in bioavailability, metabolic stability, target specificity, and determination of optimal dosing. Addressing these challenges requires further investigation into structure–activity relationships, pharmacokinetics, and potential interactions among different classes of phytochemicals [15].

This narrative review was prepared in accordance with the SANRA guidelines to ensure methodological quality and transparency. A comprehensive literature search was conducted using PubMed, Scopus, Embase, and Web of Science for articles published up to December 2025. This article provides a comprehensive analysis of how polyphenols, flavonoids, and carotenoids interact with oxidative pathways in human pathologies. The authors present an integrated view of the therapeutic potential and limitations of phytochemical antioxidant treatments based on evidence from biochemistry, cell biology, and clinical practice. The information presented will be useful for advancing the development of future redox-modifying therapies.

## 2. Oxidative Stress and Redox Signaling in Human Pathophysiology

Aerobic organisms produce reactive oxygen species (ROS) and reactive nitrogen species (RNS) as byproducts of metabolic processes, and these molecules function both as normal products of cellular activity and as signaling mediators [16]. Under normal circumstances, ROS and RNS are generated in appropriate amounts, and their levels are tightly regulated within the cellular environment. This regulation allows ROS and RNS to play important roles in cell signaling, host defense mechanisms, and adaptive responses to environmental threats [17].

However, oxidative stress occurs when there is an excessive or uncontrolled production of ROS and RNS, which overwhelms the cell’s natural defense mechanisms and results in oxidative damage, ultimately contributing to the development of various human diseases (Figure 1) [18].

### 2.1. ROS/RNS Generation and Cellular Damage

The mitochondrial electron transport chain, NADPH oxidases (NOX), xanthine oxidase, peroxisomal oxidases, and uncoupled nitric oxide synthase (NOS) are the major intracellular sources of reactive oxygen species (ROS) in cells. Superoxide anion (O_2_^−^) is produced from partially reduced oxygen during mitochondrial respiration because of electron leakage from complexes I and III of the electron transport chain [19]. The superoxide anion is quickly dismutated to hydrogen peroxide (H_2_O_2_)s by superoxide dismutases (SOD) and then can be detoxified further to water by catalase (CAT) and glutathione peroxidases (GPx). In parallel, reactive nitrogen species (RNS) such as nitric oxide (NO) are produced by NOS isoforms, including endothelial, neuronal, and inducible NOS. NO reacts with superoxide to produce peroxynitrite (ONO^−^), a powerful oxidant with the potential to damage cellular macromolecules. Oxidative modifications performed by excessive quantities of ROS and RNS occur due to their excess formation [20]. Membrane integrity is compromised by the lipid peroxidation of UFA’s due to changes in membrane fluidity caused by the presence of LNA’s (malondialdehyde and 4-hydroxynonenal). The generation of these products compounds the cellular damage caused by lipid peroxidation. Proteins may also undergo oxidative damage, resulting in Carbonylation, the oxidation of Cysteine & Methionine residues, and a disruption to both enzymatic activity and structural stability [21]. The oxidative damage done to DNA will include base modifications, strand breaking, and chromosomal instability. This DNA damage came because of the action of ROS attacking nucleic acids. The damage done to DNA contributes to Mutagenesis, genetic instability, and Carcinogenesis [22]. The above-listed damages include impairment of normal cell function and initiation of diseases/conditions such as Inflammation, Apoptosis, Senescence, and Tissue Degeneration.

### 2.2. Redox Signaling Versus Oxidative Distress

Some types of ROS/RNS are not bad when produced, especially those produced at low or moderate levels. They play a key role in the “cell signaling cascade” as second messengers for redox signaling pathways [23]. An example is hydrogen peroxide acting as a signaling molecule that will diffuse into the cell and oxidize specific amino acids (cysteine) of certain target proteins to alter their function, location, or interaction with other proteins. Redox-sensitive signaling pathways control important physiological functions, such as proliferation, differentiation, immune response, and stress response [24]. One critical set of pathways that is influenced by redox signaling is the Nrf2 pathway, which regulates how cells respond to oxidative stress and how they protect themselves from injury. Under normal circumstances, Nrf2 is trapped in the cytoplasm by its binding partner Keap1, which marks it for degradation through the proteasome [25]. However, when Keap1′s cysteines are modified by low-level oxidative stress, or when Keap1 binds to an electrophilic molecule, Nrf2 can move into the nucleus and activate the transcription of antioxidant and detoxifying enzymes (e.g., superoxide dismutase (SOD), catalase (CAT), glutathione peroxidase (GPx), heme oxygenase (HO)-1, and glutathione synthesis enzymes). Likewise, ROS can affect the activity of MAPKs, phosphoinositide-3 kinase/Akt, and NF-κB to modulate the cellular inflammatory response and enhance cell survival [26]. The difference between healthy redox signaling and unhealthy oxidative stress is determined by how much, where, and how long ROS/RNS is produced. When ROS is released for a short time in a certain spot of the cell, it provides a way for cells to communicate with each other and respond to changes in the environment [27]. However, if an abnormal amount of ROS is produced continuously or for a long period, oxidative stress occurs and is characterized by irreversible damage to macromolecules (proteins, lipids, nucleic acids, etc.) and unregulated cellular signaling [28]. The shift in the balance from healthy to unhealthy oxidative signaling is due to mitochondrial dysfunction (and a lot of times, this is caused by chronic inflammation), age, and toxins collected from the environment [29]. In this new context, oxidative stress represents an imbalance in the signaling pathways that are produced (ROS and RNS). The therapeutic goal of addressing oxidative stress is not to scavenge free radicals but rather to restore normal levels and preserve normal cell communication through redox signaling pathways [30]. This understanding of the roles of ROS and RNS in cellular physiology and pathology establishes the scientific basis for developing redox-based therapies for chronic and degenerative diseases based on the redox signaling pathway [31].

## 3. Classes of Antioxidant Phytochemicals

**Figure 2:**
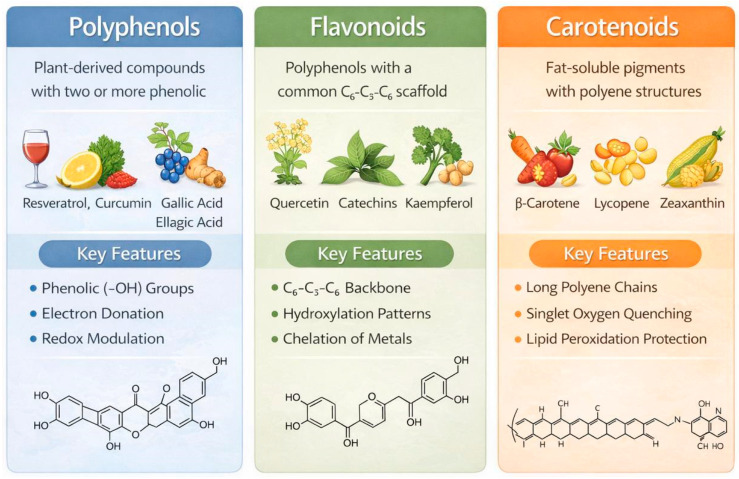
Classes of Antioxidant Phytochemicals. Created in BioRender. Singh, D. (2026) https://BioRender.com/ycuvbiq (accessed on 4 February 2025).

Phytochemicals originating from plants contain a myriad of molecular structures that can be categorized by function, which support the modulation of oxidative stress pathways and the regulation of cellular stress pathways [32]. Polyphenols, flavonoids, and carotenoids are three widely researched groups of phytochemicals, due to their high content in the human diet and their wide range of biological actions. Rather than simply acting as passive scavenging agents for free radicals, these phytochemicals are key regulators of redox homeostasis, inflammation, and metabolic signalling, suggesting that they may serve as useful therapeutic agents for diseases that result from oxidative stress, as shown in Figure 2 [33].

### 3.1. Polyphenols

The polyphenols are a large and diverse group of secondary metabolites produced by plants that contain more than one type of phenolic hydroxyl (–OH) group. The polyphenols are divided into the following classes: tannins, phenolic acids, lignans, stilbenes, etc. Some of the more well-known polyphenols include resveratrol, curcumin, gallic acid, and ellagic acid [34]. The redox activity of polyphenols is due to their ability to donate hydrogen atoms and/or electrons to reactive oxygen species (ROS), which results in the formation of a stable phenoxyl radical. In addition, by stabilising the resulting phenoxyl radical through resonance structures, polyphenols can enhance the activity of their own redox-active molecules by allowing them to act as an efficient oxidant in the presence of ROS [35]. Polyphenols also have a major regulatory role on redox-sensitive signalling pathways in the cell, and thereby activate cellular defence mechanisms via the nuclear translocation of Nrf2 (Nuclear Factor Erythroid 2, Related Factor 2). By inducing Nrf2 translocation into the nucleus and subsequently increasing the expression of detoxifying and antioxidant enzymes (such as GSTs and HO-1), polyphenols also reduce oxidative and inflammatory signalling in cells through inhibition of NF-κB, MAPK, and cyclooxygenase pathways [36]. This combined effect allows polyphenols to reduce oxidative stress and simultaneously increase cellular defence systems, making polyphenols unique compared to standard antioxidants [37]. Polyphenols are also thought to impact mitochondrial bioenergetics, autophagy, epigenetic regulation, and thus link redox regulation to longevity of a cell and its metabolic health. However, poor bioavailability and rapid metabolism can limit the therapeutic value of polyphenols; thus, there has been a resurgence of interest in the development of polyphenol formulations and analogs [38].

### 3.2. Flavonoids

Flavonoids constitute a major subclass of polyphenols, unified by a common C_6_–C_3_–C_6_ backbone and subdivided into flavonols, flavones, flavanones, isoflavones, flavanols, and anthocyanins. Widely distributed in fruits, vegetables, tea, and medicinal plants, flavonoids such as quercetin, kaempferol, catechins, and genistein exhibit potent antioxidant and cytoprotective properties [39]. Structurally, the number and position of hydroxyl groups, degree of conjugation, and glycosylation patterns critically determine flavonoid redox behavior. These features enable flavonoids to chelate transition metals, inhibit lipid peroxidation, and stabilize cellular membranes under oxidative stress. Unlike classical antioxidants, flavonoids act as redox modulators by fine-tuning intracellular signaling rather than indiscriminately eliminating ROS [40].

Polyphenols include flavonoids. Many flavonoids, such as flavones and flavanones, have a common base of C_6_–C_3_–C_6_ Flavonoids can be further broken down into many subcategories, like anthocyanins, isoflavones, flavonols, and flavanols [41]. The flavonoid content of various foods and beverages includes fruit, vegetables, tea, and other herbal supplements, with many potent antioxidants and cytoprotective agents. Quercetin, Kaempferol, catechins, and Genistein, for example, have been shown in numerous studies to exhibit powerful antioxidant and cytoprotective effects [42]. The structural characteristics of flavonoids, such as the number and position of hydroxyl (OH) groups, the degree of conjugation, and glycan antioxidants, all contribute significantly to the redox properties of flavonoids. These attributes of flavonoids allow them to chelate metal ions, inhibit lipid peroxidation, and stabilize membranes during oxidative stress [43]. Unlike traditional antioxidants, which simply remove ROS, flavonoids act as redox modulators and play a significant role in the control of intracellular signaling through precise control of oxidative stress rather than simply eliminating it [44].

### 3.3. Carotenoids

Carotenoids are fat-soluble pigments that impart the yellow, orange, and red hues of many fruits and vegetables. Examples of carotenoids include β-carotene and lycopene, which are included in the category known as carotenes; and xanthophylls (the latter two being lutein and zeaxanthin). Because they have a linear structure called polyene, carotenoids can effectively dissipate the harmful effects of singlet oxygen and neutralize oxidative free radicals formed by lipid peroxidation [45]. Therefore, carotenoids provide an incredible level of protection for cellular membranes from oxidative destruction. The chemical composition of carotenoids allows them to be more effective than polyphenols or flavonoids, which are common antioxidants, in stabilizing membrane structure and preventing lipid oxidation within membranes, because carotenoids primarily concentrate in lipid bilayers [46]. In addition to having antioxidant properties, carotenoids play an important role in regulating gene expression, supporting the immune system, and facilitating communication between cells. Some carotenoids can be converted into bioactive metabolites such as retinoids, which are known to regulate various cellular processes, including differentiation and responses to oxidative stress [47].

The importance of carotenoids to overall tissue-specific redox protection cannot be overstated, but rather it is their preferential distribution in the retina, brain, and cardiovascular system that illustrates their critical role [48]. The antioxidant properties of carotenoids can vary because of the way their environment interacts with them, where higher concentrations or exposure to reactive oxygen species may result in changes in carotenoid redox behaviour. Therefore, with respect to carotenoid-based intervention, there is a need to understand these three factors: dose, formulation, and biological context [49].

## 4. Molecular Mechanisms Targeting Oxidative Pathways

**Figure 3:**
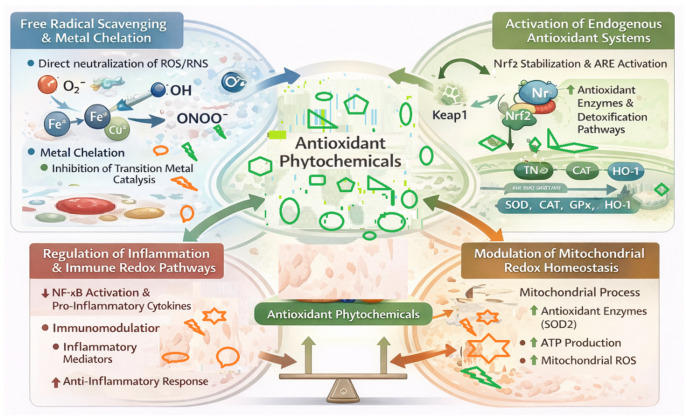
Molecular Mechanisms Targeting Oxidative Pathways. Created in BioRender. Singh, D. (2026) https://BioRender.com/yfq3a0n (accessed on 6 February 2025).

Antioxidant compounds provide health benefits by changing multiple pathways involved in oxidative stress rather than simply blocking free radicals. In addition to blocking free-radical formation, antioxidant compounds target molecules with redox (oxidation/reduction) properties and help to restore the cell to homeostatic balance, maintain proper signalling functions, and repair damage from oxidative stress caused by many chronic diseases [50]. The four major mechanisms by which antioxidant phytochemicals exert their effects include: (1) neutralizing free radicals directly; (2) activating the body’s own antioxidant systems; (3) acting on the balance of high and low energy oxidation-reduction reactions within mitochondria; and (4) controlling communication between the immune system and inflammation (inflammatory) responses (Figure 3) [51].

### 4.1. Free Radical Scavenging and Metal Chelation

The first and earliest established mechanism by which phytochemicals may serve as antioxidants is the removal of reactive oxygen and nitrogen species. Reactive oxygen and nitrogen species include free radicals such as superoxide anion, hydroxyl radical, and peroxynitrite (NO_2_−) and many other unstable species [52]. Polyphenols (and flavonoids) accomplish this by providing electrons or hydrogen atoms to positively charged free radicals and therefore stop the chain reaction of lipid peroxidation. The structural features of polyphenolic compounds provide stabilizing environments for the resulting radical intermediates [53]. In addition to the above mechanisms, many phytochemical compounds can interact with redox-active transition metals such as iron and copper and thus become less capable of generating reactive oxygen species via the Fenton and Haber–Weiss (HQ; Fe) reaction pathways [54]. As such, through this mechanism, phytochemicals decrease the oxidative burden associated with the generation of free radicals through metal-mediated processes. Finally, the chelation of iron and copper also contributes to the neuroprotective and cardioprotective function of many phytochemicals, under conditions of iron and/or copper dysregulation where increased oxidative injury occurs [55]. While the direct radical scavenging activity of phytochemicals receives significant attention as a mechanism of action, this activity is becoming more recognized as a cofactor or secondary, rather than primary, mechanism in the body, particularly during conditions associated with chronic oxidative stress [56].

### 4.2. Activation of Endogenous Antioxidant Systems (Nrf2–ARE)

One of the main mechanisms, which plays an important role in the redox properties of phytochemical substances, is not the direct scavenging of free radicals in vivo but rather the regulation of the body’s own mechanisms of protective responses to oxidative stress [57,58]. Moreover, direct scavenging is not possible for most free radicals, except peroxyl free radicals in a lipid environment [58]. Another crucial observation is the fact that hydroxyl free radicals cannot be scavenged in vivo by any antioxidant, including phytochemical substances, due to their highly diffusible characteristic of rapidly reacting with any molecule within their environment [58].

Conversely, the major antioxidant action exhibited by phytochemicals lies in their capacity to induce an adaptive cellular response pathway, specifically through the nuclear factor erythroid 2-related factor 2 (Nrf2)-antioxidant response element (ARE) pathway [57]. In non-stressed or resting conditions, Nrf2 remains localised to the cytoplasm, suppressed by Kelch-like ECH-associated protein 1 (Keap1), an inhibitor of Nrf2 that targets it to proteasomal degradation. The mild electrophilic or redox properties endowed to some phytochemicals have been shown to target specific cysteine residues on Keap1, interfering with the Keap1-catalysed ubiquitination of Nrf2, which ultimately translocates to the nucleus [58].

Once translocated into the nucleus, Nrf2 binds to ARE sequences and initiates a coordinated transcriptional programme that amplifies cellular resilience against oxidative and electrophilic insults [59]. This program involves the induction of enzymes associated with glutathione biosynthesis and recycling, for example, glutamate–cysteine ligase (GCL); the phase II detoxification, for example, NAD(P)H quinone oxidoreductase 1 (NQO1); redox homeostasis, for example, thioredoxin reductase (TRR); and cytoprotective stress responses, for example, heme oxygenase-1 (HO-1). The phytochemicals provide sustained and systems-level protection by increasing the endogenous biochemical capacity to neutralise the oxidative insults. The protection contrasts with the transient and mechanistically limited effects from direct radical scavenging [60].

Apart from redox regulation, Nrf2 activation is currently recognised to play a wider role in regulating cellular metabolism, autophagy, and proteostasis; thus, Nrf2 activation connects a wider range of stress perception to survival pathways [61]. Nevertheless, it should also be noted and emphasised that Nrf2 overactivation has significant roles in providing a survival advantage to neoplastic cells and in chemoresistance. This implies that Nrf2 activation should thus require (1) context-dependent and precisely controlled activation and repression, and (2) further innovation in finding and understanding phytochemicals used to control Nrf2 activity in a manner appropriate in terms of specificity and timing control [62].

### 4.3. Modulation of Mitochondrial Redox Homeostasis

Mitochondria serve as both a principal producer and target of reactive oxygen species. Damage caused to the redox balance of mitochondria will result in the ineffective use of oxygen when producing ATP (i.e., oxidative phosphorylation), enhance the production of reactive oxygen species (ROS) due to leakage from the mitochondria, and promote the activation of cellular apoptosis pathways [63]. Mitochondrial function and mitochondria-derived ROS can be modified by antioxidant phytochemicals that protect mitochondrial electron transport chain components, maintain structural integrity of mitochondrial membranes, and regulate antioxidant enzyme activity (e.g., manganese superoxide dismutase) within the mitochondria [64]. Antioxidant phytochemicals also improve mitochondrial biogenesis and efficiency by activating a network of signalling pathways involving AMP-activated protein kinase and peroxisome proliferator-activated receptor-γ co-activator-1α. Carotenoids, which are highly lipophilic compounds that have a high affinity to localize within both the mitochondrial and cellular membranes, also help preserve membrane integrity by preventing lipid peroxidation and/or improving the fluidity of the membrane [65]. Phytochemicals may restore redox homeostasis within the mitochondria, thus preventing the generation of oxidative amplification loops that contribute to neurodegeneration, metabolic disorders, and age-related diseases associated with oxidative stress [66].

### 4.4. Regulation of Inflammation- and Immune-Related Redox Pathways

There is some interrelationship between oxidative stress and inflammation, creating a recurring cycle that causes additional tissue damage. Phytochemicals interfere with the cycle by regulating redox-sensitive inflammatory and immune signaling pathways [67]. Phytochemicals inhibit the activation of nuclear factor-kappa B (NF-kB), which decreases the production of pro-inflammatory cytokines, chemokines, and inducible nitric oxide synthase, decreasing the production of reactive oxygen species (ROS) and reactive nitrogen species. Phytochemicals also promote immune cell polarization and influence the function of macrophages, microglia, and lymphocytes through regulation of redox-dependent signaling [68]. This results in the reduction in chronic inflammation while preserving host defence. The interactions between Nrf2 and the inflammatory pathways contribute to the antioxidant and anti-inflammatory actions of phytochemicals as a unitary process of phytochemical action [69]. Thus, these molecular pathways illustrate the potential therapeutic applications of natural antioxidants as redox modifiers and not merely scavengers of free radicals. Understanding these pathways provides the basis for the rational design of phytochemical-based interventions for oxidative stress-related diseases [70].

## 5. Cellular and Subcellular Protective Actions

Cytoprotective action of antioxidant phytochemicals is mediated through multiple levels of cellular and subcellular interaction, where oxidative stress can lead to loss of structural integrity as well as disruption of regulatory networks [71]. Polyphenols, flavonoids, and carotenoids do not work uniformly as antioxidants; they selectively protect membranes, genetic material and stress response pathways from oxidative stress to maintain cellular function during a state of oxidative stress. This protective mechanism is very important to the development of chronic degenerative disease processes, which result from cumulative molecular damage and impaired cellular repair systems (Table 1) [72]. Oxidative stress causes damage to lipid membranes due to the high levels of polyunsaturated fatty acids present in lipid membranes and their susceptibility to peroxidation. The degradation of lipid membranes by oxidative processes will cause an alteration in the fluidity of lipid membranes, an alteration in the function of the cell membrane receptors and an alteration in the cellular signalling process [73]. The inhibition of free radical formation from the oxidation of lipids by antioxidant phytochemicals plays a large part in the protective role of antioxidant phytochemicals against lipid peroxidation and provides a means for maintaining cellular viability [74]. Lipid peroxyl radicals are neutralized by carotenoids through their ability to quench singlet oxygen and through their preferential incorporation into the lipid membranes [75]. The incorporation of carotenoids into lipid membranes provides membrane stabilisation and therefore supports all the processes occurring within the mitochondria, as well as the processes occurring within synapses. The effect of carotenoids on membranes will support the separation of electrical impulses and electrical activity by stabilising the lipid membranes through quenching of free radicals [76]. The polyphenols and flavonoids, although more hydrophilic than carotenoids, do interact with lipid membranes through their affinity to associate with lipid membranes and through their affinity to associate with proteins located on the lipid membrane surfaces [77]. Polyphenols and flavonoids help inhibit the oxidative modification of cell membrane proteins and therefore help regulate all the membrane-associated enzymes located within the lipid membranes, therefore maintaining the integrity of the cellular membranes and preventing an amplification of oxidative Stress signals through all cellular compartments [78]. Due to the likelihood of damage from oxidative stress, genomic DNA is very susceptible to oxidative harm. This oxidative damage causes mutation through both chromosomal instability and the development of abnormal DNA bases [79]. The antioxidant properties of various plant compounds can be attributed to both direct and indirect means to protect against oxidative DNA damage. These antioxidant compounds decrease intracellular levels of reactive oxygen species (ROS) and subsequently reduce the amount of oxidative DNA damage (8-oxo-2′-deoxyguanosine). Antioxidants can enhance DNA repair capability through activation of various redox-sensitive signalling pathways [80]. In addition to protection by reducing DNA oxidative damage, antioxidant compounds influence gene expression and epigenetic regulation in a manner that is dependent upon oxidative stress. Oxidative stress alters the functions of DNA methyltransferases, histone-modifying enzymes and chromatin remodelers, resulting in abnormal gene expression patterns [81]. Polyphenols and flavonoids can alter epigenetic regulation by changing histone acetylation and methylation patterns and also by affecting the expression of microRNAs that regulate oxidative stress and inflammation [82]. The epigenetic effects of antioxidant phytochemicals allow for long-term alterations in cellular responses beyond simply neutralizing ROS. Therefore, because the epigenetic redox regulation alters an organism’s ability to adapt to oxidative stress, antioxidant-rich diets have the potential to have long-term benefits on disease development and longevity [83]. Under conditions of oxidative stress, the intricate balance of factors influencing a cell’s decision to survive or die is maintained through apoptosis and autophagy. Many antioxidants are thought to assist with this regulatory process by serving as chemical intermediates to facilitate crosstalk between apoptosis, senescence, and autophagy [84]. These three processes together form a triad of processes that maintain the quality of cells and homeostasis of tissues.

The activation of autophagy in a moderate manner enables cells to remove damaged components such as oxidatively modified proteins and organelles, particularly dysfunctional mitochondria [85]. Phytochemicals support the conversion of oxidised molecules to more stable forms of polyphenols through activation of AMP-activated protein kinase and the mammalian target of rapamycin (mTOR) pathways, which contribute to increased resistance against oxidative stress. Phytochemicals can also inhibit overactive or improperly functioning forms of autophagy that may contribute to cellular death [86]. In the case of apoptosis, phytochemicals have been shown to produce contradictory results. Under a condition of pathological oxidative stress, antioxidant phytochemicals inhibit excessive apoptosis by maintaining mitochondrial integrity (thereby maintaining cytochrome c), and they may regulate the expression levels of pro-apoptotic and anti-apoptotic proteins [87]. In contrast, under conditions of neoplastic or otherwise compromised cells, certain phytochemicals may selectively induce apoptosis, thereby providing chemopreventive and anticancer activities [88]. Phytochemicals are natural antioxidants and have the potential to delay cellular senescence, caused by oxidative stress (OS), through regulating OS pathways and ROS production. OS has been shown to cause cellular senescence by halting proliferation and creating a pro-inflammatory environment [89]. Therefore, phytochemicals can help to delay the onset of cellular senescence and include redox-influenced mechanisms that can be used in combination with other agents to mitigate the negative tissue effects resulting from cellular senescence. Combined with these protective effects, phytochemicals are seen as potential candidates for ongoing therapeutic interventions to support cellular health through the stabilization of cellular integrity, protection of genes, and modification of cellular responses to stressors [90].

**Table 1:** Cellular and Subcellular Protective Actions of Antioxidant Phytochemicals.

S.N.	Target Level	Protective Action	Underlying Mechanism	Representative Phytochemicals	Therapeutic Implications	Reference
1	Cellular membranes	Prevention of lipid peroxidation	Scavenging of lipid peroxyl radicals; quenching of singlet oxygen; inhibition of oxidative chain reactions	Carotenoids (β-carotene, lycopene), flavonoids (quercetin)	Preservation of membrane fluidity; protection of neuronal and mitochondrial membranes	[91]
2	Plasma membrane–protein interface	Membrane stabilization	Protection of membrane proteins and receptors from oxidative modification; regulation of membrane-associated enzymes	Polyphenols (resveratrol, curcumin)	Maintenance of signal transduction and ion transport	[92]
3	Mitochondria	Maintenance of mitochondrial redox homeostasis	Reduction in ROS leakage; stabilization of the electron transport chain; enhancement of mitochondrial antioxidant enzymes	Polyphenols (resveratrol), flavonoids (catechins)	Improved bioenergetics; prevention of apoptosis and neurodegeneration	[93]
4	Nuclear DNA	Protection against oxidative DNA damage	Reduction in ROS-induced base lesions and strand breaks; enhancement of DNA repair pathways	Flavonoids (epigallocatechin gallate), polyphenols (ellagic acid)	Reduced mutagenesis; chemopreventive effects	[94]
5	Epigenetic machinery	Redox-dependent epigenetic regulation	Modulation of DNA methylation and histone modifications; regulation of redox-sensitive transcription	Polyphenols (curcumin, resveratrol)	Long-term regulation of antioxidant and anti-inflammatory gene expression	[95]
6	Autophagic machinery	Promotion of adaptive autophagy	Activation of AMPK; inhibition of mTOR under oxidative stress	Polyphenols, flavonoids	Clearance of damaged proteins and organelles	[96]
7	Apoptotic pathways	Regulation of programmed cell death	Stabilization of mitochondrial membranes; modulation of pro- and anti-apoptotic proteins	Flavonoids, polyphenols	Cytoprotection in degenerative diseases; selective apoptosis in cancer cells	[97]
8	Cellular senescence pathways	Delay of oxidative stress–induced senescence	Reduction in chronic ROS; regulation of redox-sensitive senescence signaling	Polyphenols, carotenoids	Anti-aging effects: improved tissue homeostasis	[98]

### 5.1. Prevention of Lipid Peroxidation and Membrane Stabilization

Lipid membranes are primary targets of oxidative stress due to their high content of polyunsaturated fatty acids, which are highly susceptible to peroxidation. Oxidative degradation of membrane lipids disrupts membrane fluidity, alters receptor function, and compromises ion channel activity, ultimately impairing cellular signaling and viability [99]. Antioxidant phytochemicals exert a critical protective role by inhibiting the initiation and propagation of lipid peroxidation chains [99]. Carotenoids, owing to their lipophilic polyene structures, preferentially localize within phospholipid bilayers, where they quench singlet oxygen and neutralize lipid peroxyl radicals. This membrane-embedded antioxidant activity stabilizes bilayer architecture and preserves membrane-dependent processes such as mitochondrial respiration and synaptic transmission [100]. Polyphenols and flavonoids, although more hydrophilic, associate with membrane surfaces and lipid–protein interfaces, where they inhibit oxidative modification of membrane proteins and regulate membrane-associated enzymes. Collectively, these actions maintain membrane integrity and prevent the amplification of oxidative signals across cellular compartments [101].

### 5.2. DNA Protection and Epigenetic Redox Regulation

Genomic DNA is highly susceptible to oxidative damage; thus, base modification, chromosomal breakage, and genomic instability due to oxidative damage result in mutations that lead to cancer, accelerated aging, and cancer development [102]. The antioxidants found in phytochemicals protect DNA from oxidative damage via both direct and indirect methods. In addition, the reduction in the intracellular concentrations of reactive oxygen species (ROS) will decrease the likelihood of DNA lesions such as 8-oxo-2′-deoxyguanosine and increase the DNA’s ability to be repaired via activation of redox-sensitive pathways that promote repair processes [102].

In addition to directly protecting the structure of DNA, antioxidant phytochemicals also exert their effects on the epigenetic regulation of genes via redox-dependent mechanisms. Oxidative stress affects the activities of DNA methyltransferases, enzymes responsible for modifying histones, and the remodeling complexes that create chromatin. As a result, this can cause changes in the gene expression patterns [103]. Polyphenolic compounds (including flavonoids) can alter the epigenetic landscape of a cell by regulating histone acetylation and methylation states, and by modulating the levels of microRNAs involved in oxidative stress and inflammation. These epigenetic reprogramming events caused by phytochemical antioxidants may provide long-term reprogramming of the cellular response to oxidative stress by enhancing antioxidant defense and adaptation to stressors, in addition to immediate scavenging of ROS [104]. Thus, it is important to note that redox epigenetic regulation is an explanation of how antioxidants from diet provide protection against many of the diseases and conditions associated with healthy aging and reduced incidence of chronic disease.

### 5.3. Crosstalk with Autophagy, Apoptosis, and Senescence

The interplay between life and death survival pathways and cell-death pathways is critical for determining cellular destiny when there is an oxidative stress challenge [105]. The balance between these pathways is defined in part by the ability of antioxidant phytochemicals to help regulate the crosstalk that occurs between the pathways of apoptosis, autophagy, and senescence, which are the three elements of cellular quality control and tissue homeostasis [105].

Moderate activation of the autophagic pathway is a protective mechanism for the elimination of oxidatively-damaged proteins and organelles, especially dysfunctional mitochondria [106]. Phytochemicals stimulate the activation of adaptive autophagy through redox-sensitive pathways, including AMP-activated protein kinase (AMPK) and mammalian target of rapamycin (mTOR) [106]. This promotes the cellular resiliency to withstand ROS through the normalization of protein turnover by autophagic elimination of oxygen stress-damaged proteins and organelles. On the other hand, phytochemicals prevent excessive or ineffectively stimulated autophagy, which may ultimately trigger apoptosis [106].

In regard to apoptosis, the effects of phytochemicals are context-dependent. For example, under conditions of oxidative stress, where excessive or inappropriate apoptotic signaling may occur, phytochemicals inhibit this apoptotic pathway by stabilizing mitochondrial membranes, reducing cytochrome c release, and modulating the expression of pro- and anti-apoptotic proteins [107]. Alternatively, phytochemicals can induce apoptosis in damaged or transformed cells through specific chemopreventive and anticancer effects [107].

The physiological effects of oxidative stress led to increased senescence (growth arrest) and inflammation (decreased immunological response). Phytochemicals, through their ability to limit chronic levels of free radicals (ROSs), are believed to provide basic cellular protection from continued chronic insult and to delay the manifestation of all the detrimental consequences of cellular senescence [108]. Age-related illness and neurodegenerative diseases are just two examples of the areas where phytochemicals may provide a means of either slowing or stopping the progression of the disease through their protective actions [108].

Natural antioxidants have several unique actions that demonstrate how they serve to be much more than simply ROS scavengers [109]. By impregnating lipid membranes with antioxidant molecules, they protect the lipid structure from oxidative damage. They protect the genetic material of the cell through their membrane stabilization effect and by controlling the pathways that mediate the cellular response to oxidative stress [109]. These protective actions support the case for considering phytochemicals to be promising candidates for long-term therapeutic strategies based on redox properties [109].

## 6. Therapeutic Implications in Oxidative Stress–Driven Diseases

**Figure 4:**
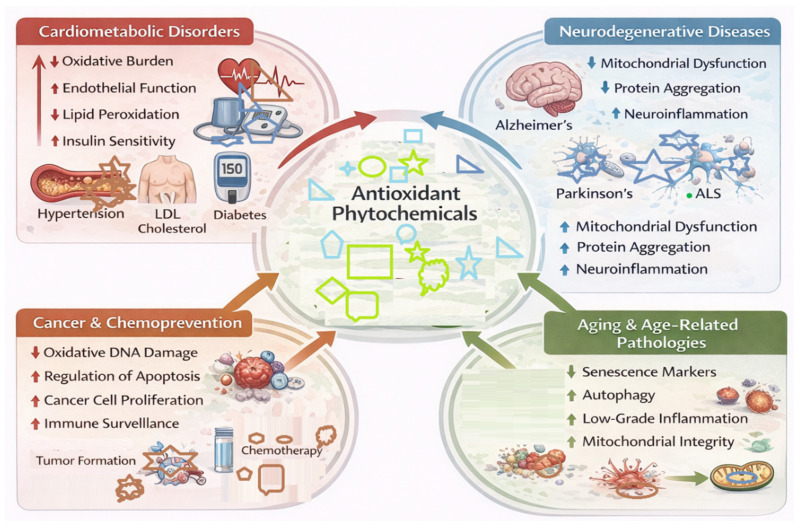
Antioxidant phytochemicals mitigate oxidative stress–driven pathologies by reducing oxidative burden, improving mitochondrial function, limiting protein aggregation and neuroinflammation, suppressing DNA damage and tumor progression, and promoting autophagy and metabolic homeostasis across cardiometabolic, neurodegenerative, cancer, and aging-related conditions. Created in BioRender. Singh, D. (2026) https://BioRender.com/6zwau7o (accessed on 4 February 2025).

Oxidative stress is a common cause of chronic disease. Sustained oxidative stress can promote tissue damage, inflammation, and reduced function of tissues (Figure 4) [110]. The use of plant-based, antioxidant-rich foods creates an opportunity to restore the balance of the various redox pathways in the body and helps strengthen the body’s ability to defend itself against material damage as well [110].

### 6.1. Cardiometabolic Disorders

Common cardiovascular metabolic conditions such as high blood pressure, hardening of the arteries, obesity, and Type 2 diabetes mellitus are associated with damaged blood vessels, inflammation, and high levels of oxidative stress (excess production of reactive oxygen species (ROS) [111]. ROS directly reduces nitric oxide availability, promotes lipids to become oxidized, and prevents insulin from working properly [111]. Dietary polyphenol and flavonoid consumption provides improvement in vascular redox balance, as they help to decrease oxidative stress on the body and increase the ability of endothelial cells to generate nitric oxide, both of which are needed to support healthy vascular tone and prevent atherogenic (plaques that form on arterial walls) conditions [111].

In addition to dietary antioxidants helping to create a redox balance, phytochemicals (naturally occurring plant substances) also positively influence how lipids are metabolized and how the body maintains glucose levels using signalling pathways that respond to changes in redox balance [112]. Phytochemicals such as carotenoids also provide a protective effect on lipoproteins by reducing oxidative modification and thus lowering the risk of foam cell formation or plaque instability. Overall improvement in metabolic health is ultimately linked to improved metabolic resilience (increased ability to manage metabolic stress) and decreased cardiovascular risk, which leads to the importance of dietary antioxidants in preventing and treating cardiovascular metabolic conditions [112].

### 6.2. Neurodegenerative Diseases

Oxidative damage occurs in neurodegenerative diseases, like Alzheimer’s, Parkinson’s, and ALS, due to the demands placed on neurons by their high metabolic activity, mitochondrial malfunction, and a lack of capacity for regeneration [113]. Oxidative stress has an impact on the aggregation of proteins, the loss of synapses, and the inflammation of the brain, which can all contribute to the progression of a neurodegenerative disease [113].

Antioxidants such as flavonoids and polyphenols (plant-derived compounds) help to protect neurons by maintaining their mitochondrial health, reducing the amount of activation of microglia (immune cells in the brain), and keeping the membrane structure and integrity of neurons intact [114]. A few of these compounds, specifically carotenoids found in higher amounts in neuronal tissue, protect the lipid-rich membranes surrounding neurons from damage caused by an excess of oxygen in the membrane. In addition, these compounds also enhance memory, attention, spatial awareness, and cognitive abilities in animals and humans and have been shown to have potential application as a treatment or preventative measure for neurodegenerative disorders [114].

### 6.3. Cancer and Chemoprevention

The effects of oxidative stress are complex in cancer biology; while they can cause genomic instability and help initiate tumours, oxidative stress is also involved in tumour progression and resistance to treatment [115]. Many phytochemicals are chemopreventive by reducing oxidative damage to DNA and controlling redox-dependent checkpoints in the cell cycle, as well as enabling the elimination of cells that are at risk of becoming malignant [115].

Both polyphenols and flavonoids can act in a context-dependent way, and they have been shown to modulate apoptosis, autophagy, and immune surveillance; they can inhibit the inflammation that promotes tumour development and selectively kill transformed cells [116]. Additionally, antioxidants can counteract the efficacy of cancer therapies that rely on the production of reactive oxygen species (ROS), highlighting the importance of dosage, timing, and patient grouping when using these phytochemicals for cancer treatment [116]. Therefore, phytochemicals may be more effective as preventative or adjunct treatments than as standalone cancer therapies [116].

### 6.4. Aging and Age-Related Pathologies

The aging process results in progressively diminishing levels of redox homeostasis, mitochondrial efficiency, and the ability of cells to repair themselves, which ultimately creates an ever-increasing accumulation of oxidative damage within cells [117]. Antioxidant phytochemicals decrease the level of oxidative stress associated with age by supporting your body’s ability to produce antioxidants and regulating its ability to enter senescence pathways [117]. Also, the modulation of autophagy, inflammation, and epigenetic processes elicits a greater resilience to stress and limits the rate of deterioration of function [118]. As well as being able to support healthy aging, the ability of antioxidant phytochemicals to reduce the level of chronic low-grade inflammation increases the risk of developing age-associated diseases, such as dementia and cardiovascular disease [118].

To summarize, the therapeutic significance of antioxidant phytochemicals lies in their ability to regulate oxidative signalling rather than to simply inhibit oxidative species [119]. Therefore, the integration of antioxidant phytochemicals into both preventative and therapeutic treatment methodologies may represent a valuable strategy for managing oxidative stress-based disease processes and the reduction in adverse effects [119].

## 7. Bioavailability, Metabolism, and Translational Challenges

Despite having major support from preclinical and mechanistic research, the clinical application of antioxidant phytochemicals has been limited [120]. Factors that hinder their ability to be implemented into medical practice include the lack of knowledge regarding the correct dose and potential side effects of both antioxidant phytochemicals, as well as their bioavailability, metabolism, and inter-individual variation in metabolism. It is necessary to develop an understanding of all factors to transition the use of antioxidant phytochemicals from a food source to a therapeutic agent (Table 2) [120].

### 7.1. Absorption, Metabolism, and Gut Microbiota Interactions

The physicochemical properties of phytochemicals, including molecular size, polarity, and lipid affinity, are critical determinants of their intestinal absorption and systemic bioavailability [133]. Most flavonoids and polyphenols are present in foods mainly as glycosylated, polymerized, or high–molecular-weight conjugates, thus poorly available for direct absorption in the small intestine. Upon intake, only a small portion is absorbed in its native form, while the majority reaches the colon, where it is subject to extensive metabolism by the gut microbiota. Both absorbed parent compounds and microbial metabolites undergo phase I and phase II biotransformation either in intestinal epithelial cells or hepatocytes; the resultant glucuronidated, sulfated, and methylated derivatives circulate systemically and may retain, reduce, or even take up new biological activities [133].

Importantly, the gut microbiota is proven to be crucial in facilitating the transformation of poorly bioavailable phytochemicals into low-molecular-weight metabolites that have improved bioavailability and unique bioactivities. For instance, it has been shown that dietary Ellagitannins do not undergo direct absorption but undergo bacterial metabolism in the colon to produce urolithins that have been shown to possess anti-inflammatory, antioxidant, and mitochondrial protective properties, among others, while having improved plasma persistence compared to their parents. Likewise, the bacterial microbiota of select individuals is shown to be able to metabolize the soy isoflavone daidzein to equol, which has improved estrogenic and antioxidant properties compared to daidzein but is confined to 30–50% of the population who have “equol-producing bacteria.” Regarding the flavan-3-ols, specifically epigallocatechin gallate (EGCG) in green tea, cocoa, and catechins in cocoa, bacterial catabolic reactions produce phenyl-valerolactones and low-molecular-weight phenolic acids that can be more easily absorbed, accounting in part for their systemic antioxidant and anti-inflammatory effects [134].

These examples demonstrate that the biological activity of phytochemicals in vivo is more likely to be mediated via their microbial metabolites than via the original compounds in foods. Thus, there is considerable individual variability in phytochemical bioavailability and pharmacodynamic response due to differences in microbial composition, enzyme activities, and functionality in the human gut. Lifestyle factors such as dietary habits, age, antibiotic use, health conditions, and genetic backgrounds all influence the structure of the human gut microbiota, thereby significantly influencing antimicrobial activities of antioxidants and phytochemicals [134]. Such a host-microbe relationship plays an important role in determining efficacy, which is often neglected in the conventional antioxidants paradigm.

Aside from activating phytochemicals, the gut microbiota is also regulated by these compounds, creating a mutually regulatory relationship. Polyphenols and flavonoids have been observed to specifically induce beneficial bacteria, including Bifidobacterium, Lactobacillus, and Akkermansia muciniphila, while inhibiting pathogenic bacteria. For example, resveratrol was observed to increase the ratio of Bacteroidetes to Firmicutes, as well as improve the integrity of the intestinal barrier, while anthocyanins specifically stimulated bacteria that produce short-chain fatty acids, which have anti-inflammatory properties that modulate the immune system. This symbiotic relationship helps to re-establish balance to the microbiome, decrease chronic inflammation, and modulate immune system function [135]. The gut microbiome not only regulates the bioactivity of phytochemicals but may also serve as a downstream target through which phytochemicals induce systemic effects.

Carotenoids, despite their different molecular structures from polyphenols, are also influenced by complex bioavailability restrictions. Due to their lipophilic properties, carotenoids need to be co-administered with lipids from dietary sources to facilitate their intestinal uptake through bile acids. Finally, carotenoids are secreted from the enterocytes through micelles and secreted from the enterocytes through micelles, which are absorbed by the cells lining the intestinal wall. Carotenoids are then transported to the systemic circulation via the lymphatic system [136]. They are distributed to different organs with a preference for their accumulation in fat-storing organs, including the liver, brain, and retina, hence playing a pivotal role as antioxidants within these cells. The bioavailability of carotenoids, however, is impaired through oxidation before or during meal intake [137].

Recent findings also provide evidence that the intestinal microbial community size and type have a downstream effect on the bioaccessibility of carotenoids, as influenced by the modulation of bile acid metabolism and intestinal epithelial permeability and the subsequent effects on micellization and epithelial transport efficacy. Additionally, the microbial metabolites of carotenoids derived from carotenoid cleavage products could be involved in redox signaling and nuclear receptor activation.

Taken together, these results highlight that rather than a silent witness, the gut microbiota plays a pivotal role as a biochemical gatekeeper in phytochemical bioavailability and efficacy. The integration concept of microbial processing in phytochemical science can thus play a critical role in enhancing mechanistic and biological insights into phytochemical science in a more precise manner. The biological factors in this host–microbiota–phytochemical nexus can thus enhance the biological basis and scope in redox biology-based antioxidant approach principles and phytochemical sciences in general.

### 7.2. Dose–Response Relationships and Safety Considerations

Antioxidant phytochemicals differ from traditional medicines in that the dose–response relationships for phytochemicals are often nonlinear and context-dependent. Within physiological or moderate doses, antioxidant phytochemicals can act to regulate the redox environment and activate internal cellular defence systems, keeping cells healthy and in balance (homeostasis) [138]. However, when consumed in high doses, some antioxidant phytochemicals may exert pro-oxidant effects on a person’s body, especially in the presence of transition metals or in a highly oxidative environment [138]. The full impact of the dose–response relationship for antioxidant phytochemicals highlights the need to optimize the dose of antioxidant phytochemicals and also questions the assumption that consuming a higher amount of antioxidant phytochemicals will provide a greater benefit [138].

In addition to the considerations mentioned above regarding the safety of antioxidant phytochemical consumption, long-term use of antioxidant phytochemicals, the use of combined antioxidant phytochemicals with other medications, and how the two interact, also complicate these safety considerations [139]. While drug manufacturers view phytochemicals as safe when they are taken as part of a normal and balanced diet, concentrated formulations (or isolated nutrients) derived from phytochemical sources may pose many safety concerns, such as Hepatotoxicity (toxicity to the liver), Disruption of the endocrine system (hormones), and Interference with the activity of drug-metabolizing enzymes [140]. In the field of oncology, there are also growing concerns surrounding the use of excessive doses of antioxidants (s) during treatment since it may negatively impact the efficacy of some ROS-dependent (reactive oxygen species) chemotherapy or radiotherapy [140]. Importantly, many of these factors are patient- or disease-specific and lead to the need for further individualized recommendations for the use of antioxidant phytochemical-based interventions, rather than recommending a single dose for everyone [141].

### 7.3. Overcoming Translational Barriers

To overcome barriers of poor bioavailability and translational issues, several new delivery systems have been created (e.g., nanoformulations, liposomal formulations, and phytochemical conjugates, etc.), which would allow for better stability and absorption of nutrients and better targeting of tissues [142]. Additionally, using phytochemicals with absorption-enhancing agents, and/or taking advantage of synergistic effects between various dietary components, may lead to increased therapeutic effectiveness with lower dosages [142].

From a clinical perspective, future trials should no longer rely primarily on simplistic antioxidant measurements for endpoint determination but instead incorporate other relevant clinical and laboratory markers such as biomarkers of redox signalling, inflammation, and metabolic adaptation [143]. Moreover, stratifying participants based on individual microbiome profiles, genetic makeup, and point of entry (stage) to their disease will enable researchers to evaluate efficacy and safety more accurately [143]. To conclude, a fundamental alteration of how people think about antioxidant phytochemicals is needed to successfully translate these nutrients to the clinic [144]. Rather than viewing them strictly as “universal” radical scavengers, it must be recognized that these compounds are biologically active modulators of cellular function, with metabolism, microbiota composition, and dose all influencing the result [144]. Addressing these issues is vital to the successful application of the full therapeutic benefit of antioxidant phytochemicals in a variety of oxidative-stress-mediated diseases [144].

## 8. Advanced Strategies to Enhance Antioxidant Efficacy

Although the biological activities of natural antioxidants are numerous, their therapeutic potential is often limited due to insufficient bioavailability, quick metabolism, and inadequate tissue distribution [145]. Researchers have developed advanced methods to improve antioxidant effectiveness by exploiting the synergistic effects of multiple phytochemicals and using new delivery methods that enhance stability, absorption, and localized action of phytochemicals. The development of these strategies will facilitate the use of redox-regulating phytochemicals in clinical practice [145] (Table 3).

### 8.1. Synergistic Phytochemical Combinations

A combination of phytochemicals working synergistically may be the most effective way to enhance their antioxidant properties while requiring less of the phytochemical to provide benefit [154]. In many biological systems, combinations of polyphenols, flavonoids, and carotenoids can attack multiple targets or nodes within oxidative and inflammatory pathways and may provide additive to supra-additive interactions [154]. For example, polyphenols that stimulate endogenous antioxidant activity, combined with carotenoids that provide membrane stabilization, can produce protective effects throughout different cellular compartments via a complementary relationship [154].

Additionally, synergy exists in the modulation of signaling pathways. Certain flavonoids will work synergistically to enhance nuclear factor erythroid 2–2-related factor 2 (Nrf2) activation and reduce nuclear factor-κB inflammatory signaling, allowing for enhanced restoration of redox balance when using these agents together vs. alone [155]. Phytochemical combinations may also enhance bioavailability and/or systemic exposure by increasing metabolic activity and/or the activity of transporters [155].

Synergistic formulations may also help to reduce the likelihood of high-dose single compound pro-oxidant effects, thereby corresponding with the hormetic characteristics of antioxidants [155]. Foods containing mixtures of different phytochemicals serve as clear examples of synergy, providing the rationale for multi-component therapeutic formulations. However, combinations must be selected based on biological mechanisms of action, pharmacokinetics, and disease-specific factors for eventual clinical translation [155].

### 8.2. Nanoformulations and Targeted Delivery Systems

Nanotechnology-based delivery systems have revolutionized the way we think about delivering antioxidant phytochemicals to the body [156]. These nanoformulations (e.g., polymeric nanoparticles, lipid nanoparticles, nanoemulsions, and solid lipid carriers) increase bioavailability through solubilization; protect bioactive compounds from chemical breakdown; and provide a controlled, sustained release of the antioxidants over time [156]. The result is a dramatic increase in how long an antioxidant remains in circulation and how well it gets into cells, allowing the antioxidant to have an increased therapeutic effect while having to use lower doses of the compound [156].

Targeted Delivery Systems provide even greater specificity in terms of delivering an antioxidant by targeting specific tissues or compartments within the cell that are under the greatest oxidative stress [157]. By adding ligands and/or antibodies to a delivery system via surface functionalization of a nanoparticle, the antioxidant(s) can be targeted to areas of inflammation (e.g., inflamed endothelial tissues), areas of tumor growth (e.g., tumors), and areas where neural tissue has been damaged due to oxidative stress [157]. For example, by delivering antioxidants specifically to mitochondria (the primary site of oxidative damage), we can successfully regulate redox within a cell and provide improved protection against mitochondrial dysfunction [158].

Nanoparticles can be engineered so that they combine the advantages provided by the different mechanisms of action of a variety of phytochemicals [159]. This allows for the delivery of phytochemical combinations with synergistic effects within the same therapeutic platform. As a result, there are opportunities to engineer nanoparticles to respond to pathological signals (for example, increasing levels of pH or reactive oxygen species), which allows for site-specific drug release and reduces the incidence of off-site toxicity [159]. Nanocarriers also provide the benefits of modifying the pharmacokinetics and biodistribution of phytochemicals without altering their inherent bioactivity [159]. There are, however, several challenges to widespread clinical implementation of nanoformulations, including issues related to the ability to scale up manufacturing, determine long-term safety, obtain regulatory approval, and provide cost-effective solutions [160]. It is important to conduct comprehensive assessments of nanoparticle biocompatibility, immunogenicity, and environmental impact to establish the translational potential of these products [160]. The development of synergistic combinations of phytochemicals and advanced methods of delivering potentially effective antioxidants represents two approaches to improving the antioxidant potential of phytochemicals. The integration of systems biology with nanomedicine represents the future of developing therapies for diseases associated with oxidative stress [161].

## 9. Clinical Evidence and Current Limitations

Since the increasing number of studies conducted to support the use of antioxidants, researchers have begun looking to support their findings with the use of human intervention studies [162]. Epidemiological research has shown that people who consume diets high in polyphenols, flavonoids, and carotenoids have a lower chance of developing chronic diseases; however, the outcomes of these studies have not been consistent or strong compared to those obtained from preclinical studies using controlled clinical trial designs [163]. This has created a great deal of confusion when attempting to apply the results of preclinical studies involving antioxidants to the many different types of human populations [163].

### 9.1. Evidence from Human Intervention Trials

Antioxidant phytochemical supplement research primarily investigates cardiac and metabolic health, cognition, inflammation, and the risk of cancer, with moderate effects reported on vascular function, lipid oxidation, glycemic regulation, and multiple inflammatory markers after supplementation with either polyphenol- or flavonoid-containing extracts, and likewise, certain interventions in cognitive contexts have led to improvements in cognitive performance or a delay in loss of functioning, especially when used with early-stage or potentially at-risk individuals [164]. Additionally, carotenoid supplementation has demonstrated a protective effect in tissue-specific environments such as retinal health and photoprotection, due to the localized accumulation of carotenoids and associated oxidative stress, reinforcing the value of the synergy of phytochemicals and their interaction with the diet rather than isolated compounds [164].

Most trials do not prove that they provide clinical benefit in many advanced diseases [165]. Because the design of each trial differs from one another (i.e., compound formulations, dosages, durations, outcomes, etc.), they are often difficult to compare directly to each other. Likewise, because surrogate biomarkers are used instead of key redox markers (the actual biological events), their results can be challenging to interpret [165].

### 9.2. Discrepancies Between Preclinical and Clinical Outcomes

There are multiple reasons for the disconnect between the hopeful promise of preclinical data and the inconsistent evidence of efficacy in clinical practice [166]. The concentrations of antioxidants used in vitro and in animals are usually at least five times greater than can be attained by oral or supplemental dietary intakes in humans [166]. The models used in both preclinical and clinical settings may not adequately reflect the complexity of human metabolism, microbiome characteristics, and variability in disease presentation and response [167].

Bioavailability and metabolic transformation also reduce the efficacy of antioxidants in clinical practice [168]. Many of the phytochemicals from which antioxidants are derived have undergone extensive metabolism and may have circulating metabolites that are vastly different from the parent phytochemical used in experimental studies [168]. This high degree of interindividual variation in gut microbiota composition, the presence of genetic and environmental factors that affect metabolic activity and responses, and the effects of lifestyle choices make the trial design process much more complex than is usually considered [169].

Interventions have different effects when they occur. Antioxidants appear to work best when applied before the onset of disease, whereas late intervention provides limited or adverse effects [170]. In addition to the time of application, indiscriminate use of antioxidants could potentially inhibit redox signaling or the mechanisms by which ROS are used by direct approaches, particularly in oncology [170]. Although there is substantial support for antioxidant phytochemicals through clinical evidence, there remain significant impediments to their clinical application that must be addressed in order to convert the excitement generated by experimental studies into concrete clinical outcomes [171]. Future clinical studies should focus on developing bioavailability-focused formulations of antioxidant phytochemicals, developing bioavailability-focused selection/exclusion criteria for study participants, characterizing the microbiome of study participants, and utilizing endpoints that are mechanistically linked to the mechanisms by which the phytochemicals exert their benefits [171]. Addressing these challenges is critical if the scientific gap between successful experimental results and successful clinical outcomes is to be bridged [171].

## 10. Future Perspectives

Growing evidence that oxidative stress functions as a dynamic regulator of cellular signaling, rather than merely a source of molecular damage, has fundamentally reshaped how the therapeutic properties of antioxidant phytochemicals are understood. This paradigm shift is particularly relevant for polyphenols, flavonoids, and carotenoids, which are increasingly recognized as redox modulators capable of fine-tuning endogenous defense systems, metabolic pathways, and inflammatory responses rather than acting as simple radical scavengers [172].

Among polyphenols, compounds such as resveratrol (from grapes), curcumin (from turmeric), and ellagic acid (from berries and nuts) have been shown to activate Nrf2-dependent antioxidant response elements (ARE), suppress NF-κB-mediated inflammatory signaling, and improve mitochondrial redox balance [58,59,60,61,62]. Notably, many of these effects are not solely attributable to the parent compounds but also to microbial-derived metabolites (e.g., urolithins from ellagitannins and phenyl-γ-valerolactones from flavan-3-ols), which exhibit enhanced bioavailability and prolonged systemic activity [58,59,60,61,62,63,64,65,66,67,68,69,70,71,72]. These observations reinforce the concept that host–microbiota co-metabolism critically shapes the in vivo efficacy of polyphenol-rich foods and extracts [58,59,60,61,62,63,64,65,66,67,68,69,70,71,72].

Flavonoids, including flavonols (e.g., quercetin), flavan-3-ols (e.g., epigallocatechin gallate [EGCG]), and flavanones (e.g., naringenin), demonstrate both direct redox-regulatory activity and indirect signaling effects [39]. Quercetin and EGCG, for instance, have been shown to suppress NADPH oxidase–driven reactive oxygen species generation, enhance endothelial nitric oxide bioavailability, and induce cytoprotective genes via Nrf2 activation [40]. While isolated flavonoids exhibit strong mechanistic effects in vitro, clinical outcomes often appear more robust when these compounds are consumed as part of synergistic phytochemical matrices, such as green tea, cocoa, and citrus fruit extracts [43]. These matrices may improve compound stability, modulate absorption kinetics, and facilitate multi-target pathway engagement, highlighting the importance of whole-extract formulations in translational applications [44].

Carotenoids, including β-carotene, lycopene, lutein, and zeaxanthin, represent a mechanistically distinct class of redox-active phytochemicals that primarily function within lipid microenvironments [45]. These compounds quench singlet oxygen, inhibit lipid peroxidation chain reactions, and influence membrane redox dynamics. Beyond their physicochemical antioxidant properties, carotenoids and their oxidative cleavage products (e.g., apo-carotenals and retinoids) act as redox-sensitive signaling molecules capable of modulating transcriptional networks, including Nrf2, retinoic acid receptors, and peroxisome proliferator-activated receptors (PPARs) [46]. For example, lycopene supplementation has been associated with reduced oxidative DNA damage and improved vascular function, whereas lutein and zeaxanthin show preferential accumulation in retinal and neural tissues, supporting tissue-specific redox homeostasis [47].

These mechanistic insights argue strongly against the continued use of broad-spectrum antioxidant supplementation and instead support a shift toward precision-targeted interventions tailored to an individual’s redox state and disease context [172]. The emerging field of precision nutrition provides a conceptual framework for optimizing phytochemical efficacy by integrating individual variability in genetics, metabolism, gut microbiome composition, and lifestyle factors [173,174]. Through the application of multi-omics technologies (e.g., genomics, metabolomics, and microbiome profiling), individuals can be stratified based on their redox phenotype and oxidative risk profile, enabling the rational design of personalized dietary or supplemental strategies [174].

Importantly, the principle of redox hormesis emphasizes that both the timing and dosage of antioxidant phytochemicals must be carefully considered [175]. For example, low to moderate doses of polyphenols such as resveratrol and curcumin may enhance adaptive stress responses via Nrf2 and AMPK signaling, whereas excessive dosing could blunt physiological redox signaling required for cellular adaptation. Thus, future antioxidant therapies should not aim to indiscriminately suppress reactive species but instead seek to preserve physiological redox signaling while inhibiting pathological oxidative amplification [176]. This adaptive antioxidant paradigm aligns more closely with the biological reality of redox-regulated cellular processes.

For the successful integration of phytochemical antioxidants into modern medical practice, these compounds must be repositioned within a framework of evidence-based therapeutic medicine [177]. This transition requires the establishment of standardized bioactive formulations, rigorous controlled clinical trials with mechanism-driven endpoints, and clear regulatory distinctions between dietary supplements and therapeutic agents. Advances in formulation science—such as nanoparticle delivery systems, lipid-based carriers, and synergistic compound combinations—offer practical solutions to longstanding challenges related to poor bioavailability and limited tissue targeting of isolated phytochemicals [178]. For instance, curcumin–piperine combinations and nano-encapsulated EGCG formulations demonstrate substantially improved systemic exposure and therapeutic consistency.

In terms of translational applications, the likely role of antioxidants from plants would be as supplements, not as drugs, but as complementary agents alongside conventional pharmacological therapies [179]. Polyphenol-containing supplements, flavonoids, and carotenoids may help increase the tolerance of drugs, thereby decreasing the side effects, as well as contribute positively to managing chronic diseases by modulating the oxidative pathway and the inflammatory pathway, which are closely related. In fact, studies conducted on processed açaí pulp extract, Euterpe oleracea, recently showed that a phytochemical matrix rich in anthocyanins, flavonoids, and phenolic compounds enhanced considerable protection against oxidative stress not only on cardiomyocytes but on an animal model as well, thereby confirming the therapeutic potential of whole matrix formulations as opposed to isolating compounds [179,180]. Indeed, the fact that the biological effectiveness of phytochemical compounds is enhanced through the combined action of multiple matrix compositions is an ever-Increasing realization, thereby lending legitimacy to a nutraceutical combination-based therapeutic rationale as proposed above. Lastly, the successful clinical translation of antioxidant phytochemicals will demand inter-disciplinary collaboration among nutritional scientists, clinicians, pharmacists, and regulatory authorities [180]. The importance of inter-disciplinary approaches lies in integrating mechanistic investigations, clinical validations, formulation, and regulatory processes. Therefore, the aforementioned approaches will help in the effective use of polyphenols, flavonoids, and carotenoids in precision therapeutic approaches based on the redox-modulating potential of these compounds, without any unintended biological effects.

## 11. Conclusions

In summary, phytochemicals with antioxidant properties are positioned at the confluence of nutrition and health and provide a flexible and biologically sophisticated means to undertake redox-based therapeutic approaches across a large range of diseases. Polyphenols, flavonoids, and carotenoids have multiple roles, including acting as “radical” scavengers, but are also dynamic modulators of redox-signalling pathways, mitochondrial functions, inflammation, and cellular responses to stress. As our understanding of the mechanism of action has improved, so too has our understanding of how these compounds help to restore redox homeostasis, while still allowing for preservation of physiological signalling, which is important for both disease prevention and therapeutic resilience. To maximize effectiveness and minimize adverse effects, the combination of approaches to precision nutrition based on genetic, metabolic, and microbiome profiles will be necessary. Also, advances in the science of formulation, targeted delivery systems, and the use of combinations of synergistic phytochemicals will help to circumvent the long-standing barriers to the translation of these products to clinical use.

## Figures and Tables

**Table 2:** Bioavailability, Metabolism, and Translational Challenges of Antioxidant Phytochemicals.

S.N.	Aspect	Key Features	Mechanistic Basis	Translational Challenges	Potential Solutions/Strategies	Reference
1	Oral Bioavailability	Generally low to moderate	Poor aqueous solubility; instability in the gastric environment; limited intestinal permeability	Subtherapeutic systemic concentrations	Nanoformulations, lipid-based carriers, cyclodextrin inclusion	[121]
2	Intestinal Absorption	Passive diffusion and transporter-mediated uptake	Dependence on molecular size, polarity, and glycosylation	Interindividual variability	Structural optimization; food-matrix–guided delivery	[122]
3	First-Pass Metabolism	Extensive phase I/II metabolism	Glucuronidation, sulfation, and methylation in the intestine and liver	Reduced parent compound availability	Use of active metabolites; prodrug approaches	[123]
4	Metabolic Transformation	Conversion to conjugated metabolites	UGTs, SULTs, and COMT enzyme activity	Unclear biological activity of metabolites	Metabolite-focused pharmacology	[124]
5	Gut Microbiota Interaction	Microbial biotransformation	Deglycosylation, ring cleavage, reduction	Variability due to microbiome diversity	Personalized nutrition; microbiome modulation	[125]
6	Tissue Distribution	Selective tissue accumulation	Lipophilicity and protein binding	Limited CNS penetration for many compounds	Targeted delivery systems; BBB-permeable formulations	[126]
7	Plasma Half-Life	Short circulation time	Rapid clearance and conjugation	Frequent dosing required	Sustained-release formulations	[127]
8	Dose–Response Relationship	Non-linear, hormetic effects	Beneficial at low doses; adverse at high doses	Inconsistent clinical outcomes	Precision dosing; redox-context–specific 9therapy	[128]
9	Safety and Toxicity	Generally safe at dietary levels	Pro-oxidant effects at high concentrations	Risk of off-target effects	Controlled dosing and long-term safety studies	[129]
10	Drug–Nutrient Interactions	Potential interaction with therapeutics	Modulation of drug-metabolizing enzymes	Interference with chemotherapy or ROS-based therapies	Timing-based administration; patient stratification	[130]
11	Clinical Translation	Limited robust clinical evidence	Variability in formulation, dose, and study design	Poor reproducibility across trials	Standardized formulations and biomarkers	[131]
12	Regulatory Challenges	Nutraceutical–drug classification gap	Lack of a unified regulatory framework	Limited therapeutic claims	Clear regulatory pathways and clinical validation	[132]

**Table 3:** Advanced Strategies to Enhance the Efficacy of Antioxidant Phytochemicals.

S.N.	Strategy	Approach/Technology	Mechanistic Advantage	Therapeutic Relevance	Key Challenges/Limitations	Reference
1	Synergistic phytochemical combinations	Rational combinations of polyphenols, flavonoids, and carotenoids	Multi-target modulation of oxidative and inflammatory pathways; enhanced Nrf2 activation with concurrent NF-κB suppression	Improved efficacy at lower doses; reduced pro-oxidant risk; mimics whole-diet effects	Complexity in optimization; variability in interactions; standardization issues	[146]
2	Whole-food matrix–based formulations	Delivery within natural food matrices or extracts	Improved absorption and stability via natural cofactors; enhanced bioactivity through matrix effects	Better clinical consistency compared to isolated compounds	Batch variability; difficulty in dose precision	[147]
3	Nanoformulations	Polymeric nanoparticles, lipid nanoparticles, nanoemulsions, and solid lipid carriers	Enhanced solubility, stability, and cellular uptake; controlled and sustained release	Increased bioavailability; reduced dose requirement; improved therapeutic index	Long-term safety, regulatory, and scalability concerns	[148]
4	Targeted delivery systems	Ligand- or antibody-functionalized nanocarriers	Site-specific accumulation in diseased tissues (e.g., brain, tumor, inflamed endothelium)	Enhanced efficacy with minimal off-target effects	Target specificity; immunogenicity risk	[149]
5	Mitochondria-targeted antioxidants	Conjugation with mitochondrial-targeting moieties	Direct modulation of mitochondrial ROS generation and redox signaling	Neuroprotection, cardioprotection, and anti-aging applications	Limited clinical validation	[150]
6	Stimuli-responsive delivery systems	pH-, redox-, or enzyme-sensitive nanocarriers	On-demand release in oxidative or inflammatory microenvironments	Precision therapy with reduced systemic exposure	Design complexity; translational feasibility	[151]
7	Microbiota-informed formulations	Prebiotic–phytochemical combinations	Enhanced biotransformation into bioactive metabolites; reduced interindividual variability	Improved systemic antioxidant effects; gut–systemic axis modulation	Individual microbiome differences	[152]
8	Adjunctive combination therapy	Co-administration with standard pharmacological agents	Reduction in oxidative side effects; improved treatment tolerance	Supportive therapy in chronic diseases and oncology	Potential drug–nutrient interactions	[153]

## Data Availability

No new data were created or analyzed in this study. Data sharing is not applicable to this article.
